# Identification of miRNA–mRNA–TFs regulatory network and crucial pathways involved in asthma through advanced systems biology approaches

**DOI:** 10.1371/journal.pone.0271262

**Published:** 2022-10-20

**Authors:** Noor Ahmad Shaik, Khalidah Nasser, Arif Mohammed, Abdulrahman Mujalli, Ahmad A. Obaid, Ashraf A. El‐Harouni, Ramu Elango, Babajan Banaganapalli

**Affiliations:** 1 Department of Genetic Medicine, Faculty of Medicine, King Abdulaziz University, Jeddah, Saudi Arabia; 2 Princess Al-Jawhara Al-Brahim Center of Excellence in Research of Hereditary Disorders, King Abdulaziz University, Jeddah, Saudi Arabia; 3 Department of Medical Laboratory Sciences, Faculty of Applied Medical Sciences, King Abdulaziz University, Jeddah, Saudi Arabia; 4 Department of Biology, College of Science, University of Jeddah, Jeddah, Saudi Arabia; 5 Department of Laboratory Medicine, Faculty of Applied Medical Sciences, Umm Al-Qura University, Mecca, Saudi Arabia; Vellore Institute of Technology, INDIA

## Abstract

Asthma is a life-threatening and chronic inflammatory lung disease that is posing a true global health challenge. The genetic basis of the disease is fairly well examined. However, the molecular crosstalk between microRNAs (miRNAs), target genes, and transcription factors (TFs) networks and their contribution to disease pathogenesis and progression is not well explored. Therefore, this study was aimed at dissecting the molecular network between mRNAs, miRNAs, and TFs using robust computational biology approaches. The transcriptomic data of bronchial epithelial cells of severe asthma patients and healthy controls was studied by different systems biology approaches like differentially expressed gene detection, functional enrichment, miRNA-target gene pairing, and mRNA-miRNA-TF molecular networking. We detected the differential expression of 1703 (673 up-and 1030 down-regulated) genes and 71 (41 up-and 30 down-regulated) miRNAs in the bronchial epithelial cells of asthma patients. The DEGs were found to be enriched in key pathways like IL-17 signaling (KEGG: 04657), Th1 and Th2 cell differentiation (KEGG: 04658), and the Th17 cell differentiation (KEGG: 04659) (p-values = 0.001). The results from miRNAs-target gene pairs-transcription factors (TFs) have detected the key roles of 3 miRs (miR-181a-2-3p; miR-203a-3p; miR-335-5p), 6 TFs (TFAM, FOXO1, GFI1, IRF2, SOX9, and HLF) and 32 miRNA target genes in eliciting autoimmune reactions in bronchial epithelial cells of the respiratory tract. Through systemic implementation of comprehensive system biology tools, this study has identified key miRNAs, TFs, and miRNA target gene pairs as potential tissue-based asthma biomarkers.

## Introduction

Respiratory diseases such as asthma, chronic obstructive pulmonary disease (COPD), and coronavirus have become a global concern due to the increased morbidity and mortality rates associated with them [1–3]. Asthma is a major non-communicable, life threatening, chronic inflammatory lung disease, that is characterized by repeated obstruction of the airways and airway hyper-responsiveness (AHR). In asthma, bronchial tubes secrete extra mucus, making breathing very difficult [4]. In 2019, around 262 million people were affected, and an estimated 461,000 deaths occurred due to asthma [5]. People of varied age groups are affected by asthma, which is often believed to start during childhood [6]. Asthma can be mild to severe based on the degree of clinical symptoms like mild cough, shortness of breath, tightening of the chest, and wheezing. Dust, cold air and exposure to pollen can trigger asthma attacks in affected patients [2, 7]. Both adaptive and innate immune systems are known to play an important role in asthma pathogenesis [8]. The therapeutic treatment of asthma involves the use of corticosteroid inhalers, bronchodilators, leukotriene modifiers and anti-E, anti–IL5, anti–IL4/IL13 antibodies, coupled with lifestyle modifications to reduce the exposure to environmental allergens [9].

The molecular pathogenesis of asthma is complex due to the involvement of multiple genetic, physiological, and environmental factors [10]. The large scale genome-wide association studies (GWAS) conducted on asthma patients, have highlighted the contribution of many single nucleotide polymorphisms (SNPs) in genes that are known to be expressed predominantly in the bronchial epithelial and immune cells (TSLP, IL33, GSDMB, IL1RL1 and ADAM33) [10]. Gene expression plays a pivotal role in controlling various cellular functions, including cellular growth, differentiation, inflammation, cell death, and immune function. Gene expression studies of asthmatic people have provided evidence that transcriptomic changes are crucial to initiating or promoting the cascade of immune reactions [11].

MicroRNAs (miRNA) are a small and conserved class of 18–25 nucleotide long noncoding RNA molecules that regulate post transcriptional gene expression by controlling mRNA degradation or translational repression. Over the last decades, miRNAs have emerged as potential diagnostic and therapeutic biomarkers for different complex diseases like cancers, cystic fibrosis, -thalassemia, and Duchenne muscular dystrophy [12].

Altered mRNA and miRNA expressions have been widely observed in asthma conditions [10, 13]. For example, gene expression studies have identified differential expression of chemokine (C‐X‐C motif) receptor 2 (CXCR2); alkaline phosphatase isozyme (ALPL); Charcot‐ Leyden crystal protein (CLC); carboxypeptidase A3 (CPA3); deoxyribonuclease I‐like3 (DNASEIL3) and IL‐1β (IL1B) in asthma pathogenesis [14, 15]. Similarly, several miRNAs, such as miR-27b-3p, miR-513a-5p, miR-22-3p, miR-19a, miR-133a, miR-221, miR-3162-3p, that regulate both adaptive and innate immune systems via expression of key genes that play a pivotal role in the pathogenesis of asthma have been reported. Additionally, miR-148a, miR-148b, and miR-152 targeting HLA-G, an asthma susceptibility gene, are also widely reported [16] as the contributors. However, the molecular cross talk between the mRNA-miRNA expression changes is notwell characterised.

A better understanding of the factors contributing to changes in gene expression is vital in deciphering the detailed molecular pathology of asthma or other complex diseases. Various differential gene expression studies have been conducted in asthma, but a clear understanding of the molecular function has not yet been achieved. Given the close functional association of miRNAs and mRNAs in regulating various cellular functions and biological processes, we believe that understanding the interactions between these two classes of RNAs may provide further insights into the pathophysiology of asthma. last two decades have witnessed the power of computational biology methods in studying global expression changes from diverse range of human tissues and diseases. Lack of extensive information from the literature has led us to examine the shared differentially expressed miRNAs (DEMiRs) and their target genes (DEGs) between normal and asthmatic tissues of adults to identify potential asthma biomarkers by employing robust bioinformatic gene network analysis and advanced statistical tools.

## Materials and methods

### Data curation

The gene expression omnibus (GEO) database was data source for collecting the public domain transcriptomic datasets. We downloaded two mRNA datasets, of which, GSE43696 expression series consisted of transcriptomics data of bronchial epithelial cells collected from 38 severe refractory asthma patients and 20 healthy control samples [17] generated on Agilent Human GE 4×44K V2 Gene Expression microarrays. The second series, GSE64913, consisted of transcriptomics data of epithelial brushings obtained from severe asthma patients (N = 17) and healthy volunteers (N = 23) [18], which was generated on Affymetrix HG U133 plus 2.0 GeneChips. The GSE25230 dataset consists of the miRNA expression profiles of human bronchial epithelial cells from seven healthy donors and seven asthma patients [19] generated on the Affymetrix microarray platform. The details of clinical characteristics of the subjects, sample collection, processing, total RNA isolation, and microarray steps can be found in the corresponding research articles cited above [17–19].

### Identification of DEGs and DEMiRs

Differentially expressed genes (DEGs) between asthma and healthy control samples were analysed with the Bioconductor package in the R program. The raw expression datasets were processed with R. The raw intensity signals of the expression data were uploaded into the Bioconductor-Affy package to standardize and reduce the data noise. The median values of raw signal intensities were standardized to baseline using the Robust Multichip Average (RMA) algorithm [20]. The student t-test was used to calculate statistically significant DEGs between normal and asthmatic samples. Benjamini and Hochberg’s false discovery rate (FDR) was set at p = <0.05 to select key genes and to eliminate false positive data [21].

### Functional annotation of DEGs

The DEGs (log2 fold change > 1; FDR 0.05) were functionally analysed using ClueGO 2.2.6 version [22] and CluePedia 1.2.6 [23]version. ClueGO investigates the distribution of target genes across Gene Ontology (GO) terms and pathways to create the annotation network. CluePedia is a Cytoscape plugin that provides pathway insights by combining experimental and *in-silico* data [23]. CluePedia, a ClueGO plugin, performs linear and non-linear statistical calculations from experimental data to find new biomarkers from the pathway data [22]. The P value was calculated in the ClueGO tool using right-sided hypergeometric tests with Benjamini-Hochberg adjustment for multiple test correction [22]. A statistically significant deviation from the expected distribution was indicated by an adjusted P value of 0.001, and the corresponding GO terms and pathways were enriched for the target genes. ClueGO [22] was used to calculate the strength of the association between the terms, using a corrected kappa statistic score threshold of 0.4. Similarly, relationship between the selected terms was defined based on their shared genes. The GO terms were represented as nodes in the network, and the size of the nodes reflected their enrichment significance. The network was generated automatically using the organic layout algorithm supported by the Cytoscape [24]. The visualized network functional groups were represented by their most significant terms and provides an insightful view of their interrelationships [25]. The DEGs were then enriched with different GO terms of biological process (BP), molecular function (MF), and cellular component (CC), as well as KEGG pathways.

### Identification of MiRNA target genes

The R package multiMiR was employed to retrieve the DEmiR target genes. It contains a wide collection of validated and predicted miRNA–target interactions and their associations with drugs and diseases. It is composed of murine and human datasets from 14 external databases, which include three validated, eight predicted, and three drug- or disease-related miRNA–target databases [26]. Of these, we only considered three databases (miRecords, miRTarBase, and TarBase) for analysis of the predicted target genes [27].

### Construction of miRNA-mRNA network

We have used the miRNet online tool [28] to predict and construct the miRNA-mRNA cluster network. The miRNet constructs networks miRNA-protein coding genes, miRNA-lncRNA, miRNA-circRNA, and miRNA-sncRNA, and supporting the statistical and functional interpretation of the data [29]. Furthermore, we used the starBase tool to identify the competitive endogenous RNAs [30]. The miRNA-mRNA co-expression interactions network was constructed using CoMeTa [31] and miRSig [32].

### Identifying the miRNA-target gene and TFs

Post-transcriptional regulation of gene expression is executed by miRNAs [33], while transcription factors (TFs) play a pivotal role in activation or repression of the transcription rate at the pre-transcriptional stage [34]. Hence, we employed the Cytoscape 3.7.1 tool to visualize the interactions between miRNAs and their potential target genes to understand the miRNA–target regulatory network.

### Regulatory network analyses of miRNAs-TFs-target genes

TFs were identified using the iRegulon computation tool in the gene sets of DEGs and the target DEGs. iRegulon is a database of approximately 10,000 TF motifs, and is used to detect the enriched TF motifs in the regulatory regions around each gene. Each candidate TF is linked to enriched TF motifs and is used to identify the appropriate subset of the direct target genes. In Cytoscape, iRegulon (ver. 1.3, http://apps.cytoscape.org/apps/iRegulon) [35] plugin was used to analyse and predict TF-target gene interaction pairs in the PPI network. The TF-target interaction networks with a Normalized Enrichment Score (NES) of  > 4 were selected for downstream analysis. Then, we used the over representation enrichment analysis (ORA) method to predict the miRNAs-target genes. Threshold settings of count number ≥ 2 and p value of < 0.05 were applied. Finally, the Cytoscape was used to construct the miRNAs-TFs-target regulatory network [36].

### Binding interaction of miRNA-target genes

The RNA hybrid tool (https://bibiserv.cebitec.uni-bielefeld.de/rnahybrid/) was used to find the binding affinity between the miRNA and its target gene using the minimum free energy hybridization method. This is executed in domain mode, where short RNA sequences are hybridized to the best fitting pose of the longer RNA sequences. This webserver is primarily built to predict miRNA target genes. Initially, sequences of miRNA and its target genes (3´-UTR, 5´-UTR, and coding sequences) were retrieved from the Ensembl genome browser (https://asia.ensembl.org/index.html). The default parameters used in the analysis were as follows; helix constraint of 2–8, no G: U in seed sequence, and >-18 kcal/mol of minimum free energy threshold. Seed complementarity and high base-pairing stability were considered for reducing the false positive predictions.

## Results

### Identification of DEGs

**Fig 1:**
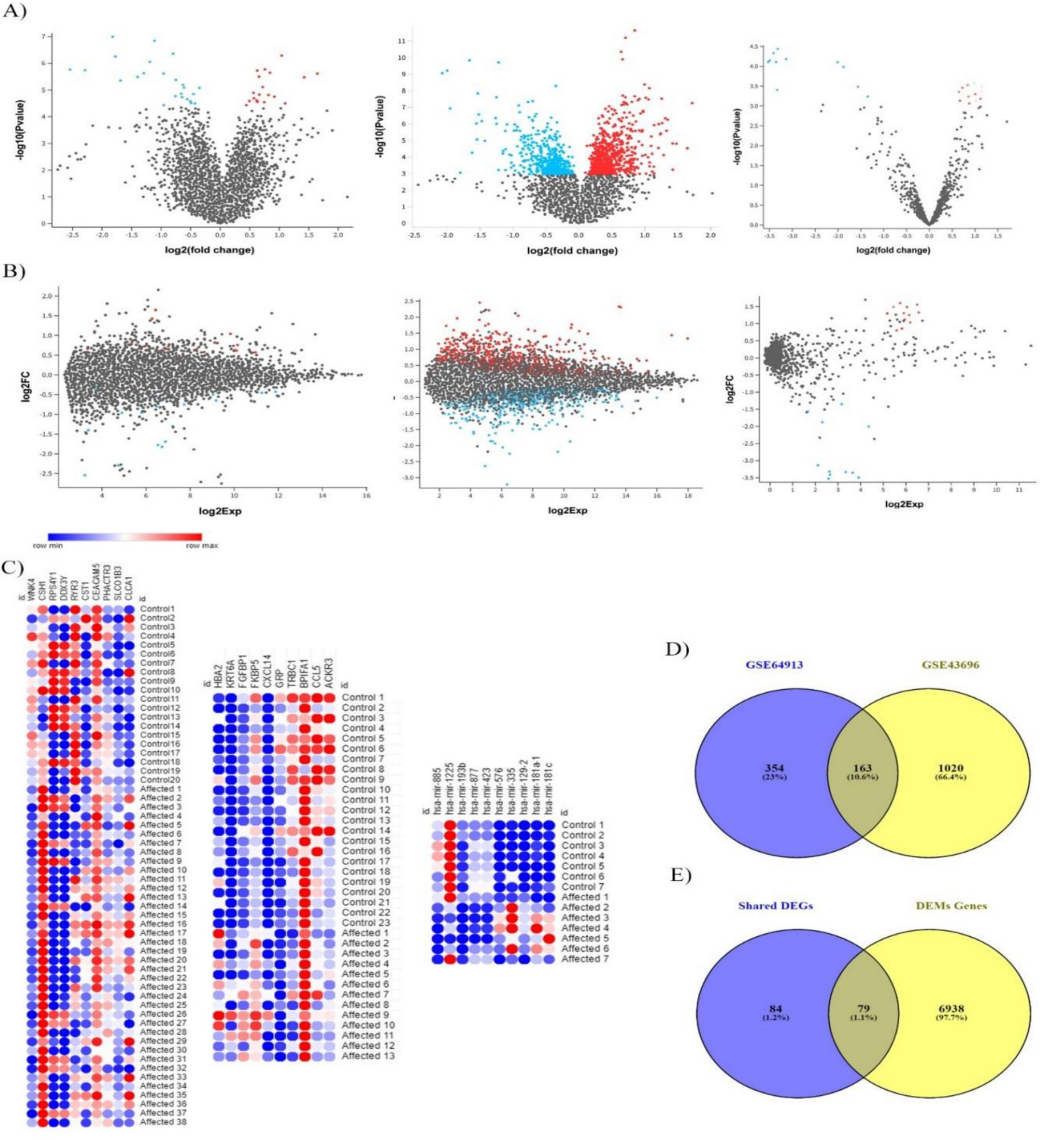
The mRNA and miRNA expression analysis of asthma patients. a) volcano plots for three differential gene expressions in 3 datasets (2mRNA and 1miRNA). Scattered points represent genes: the x-axis is the fold change for the ratio of control vs asthma, whereas the y-axis is the -log10 or P-value. Colored dots are the significantly differentially expressed genes. b) Mean difference (MD) plot showing log_2_-fold change versus average log_2_ expression values of differentially expressed mRNA or miRNA (significantly expressed mRNA or miRNA highlighted with colored dots). c) Heat map of top significant expressed mRNA and miRNA represented by red and blue color, respectively; white color indicates the median level (Generated from https://software.broadinstitute.org/morpheus) d) Venn plot representing shared gene in two datasets (mRNA) and shared miRNA target genes from one dataset.

The differentially expressed genes were identified across all the three expression datasets. Two of which are comprised of mRNA (GSE64913, GSE43696) and one is miRNA (GSE25230) data (see methods for details). The mRNA expression profile revealed the upregulation of 673 genes (258 genes in GSE64913 and 412 genes in GSE43696) and the downregulation of 1030 genes (259 genes in GSE64913 and 771 genes in GSE43696). The volcano plots and heatmap of DEGs are illustrated in **Fig 1A–1C**. A total of 163 overlapping genes (both up- or down- regulated) were identified across these two datasets, as shown in the Venny plot (**Fig 1D**). In the third dataset (GSE25230), a total of 71 differentially expressed miRNAs (DEMs), including 41 up and 30 down- regulated miRNAs, were identified.

### The functional enrichment analysis of DEGs

**Fig 2:**
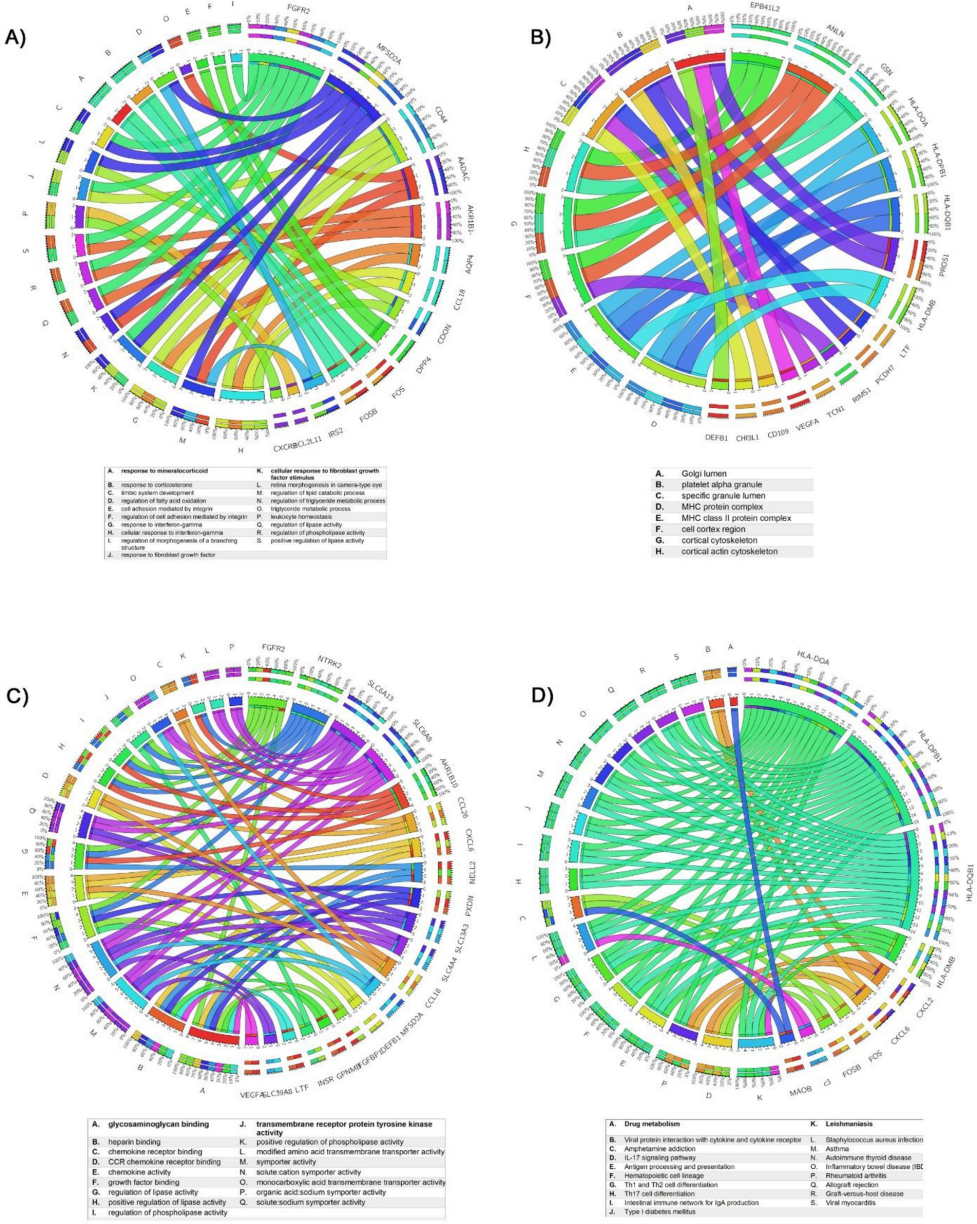
The Circos plot representation of GO-Annotation terms of differentially expressed genes. a) biological process b) cellular components c) molecular function d) KEGG pathways (Cricos plot generated from http://mkweb.bcgsc.ca/tableviewer/).

To provide further insights into the mechanism and the functional significance of these DEGs, we used the ClueGO plugin, as detailed in the methods section. The enrichment of DEGs under different GO categories like molecular function (MF), cellular component (CC), and biological process (BP) in addition to the KEGG pathways was analyzed using Circos plot representation, keeping the p-value < 0.05 as the threshold significance value (Fig 2). In the BP-associated category, the most significantly enriched GO terms were exocrine system development (GO: 0035272) with a p-value = 0.0118, response to mineralocorticoid (GO: 0051385) with a p-value = 0.0007, cell adhesion mediated by integrin (GO: 0033627) with a p-value of 0.000145, and in the cellular component category, most of the DEGs were localized in the Golgi lumen, platelet alpha granule (GO: 0005796, GO:0031091) with a p-value of <0.005. Under the molecular functions category, most DEGs were enriched in phosphatidylinositol 3-kinase binding, protein phosphatase 1 binding, and chemokine activity (GO: 0043548, GO: 0008157, GO: 0042379) with a p-value of <0.00014. The DEGs were found to be enriched in key pathways like IL-17 signaling (KEGG: 04657), Th1 and Th2 cell differentiation (KEGG: 04658), and the Th17 cell differentiation (KEGG: 04659), with a p-value of <0.00025 (Fig 2).

### Computational evaluation of miRNA enriched target genes

**Fig 3:**
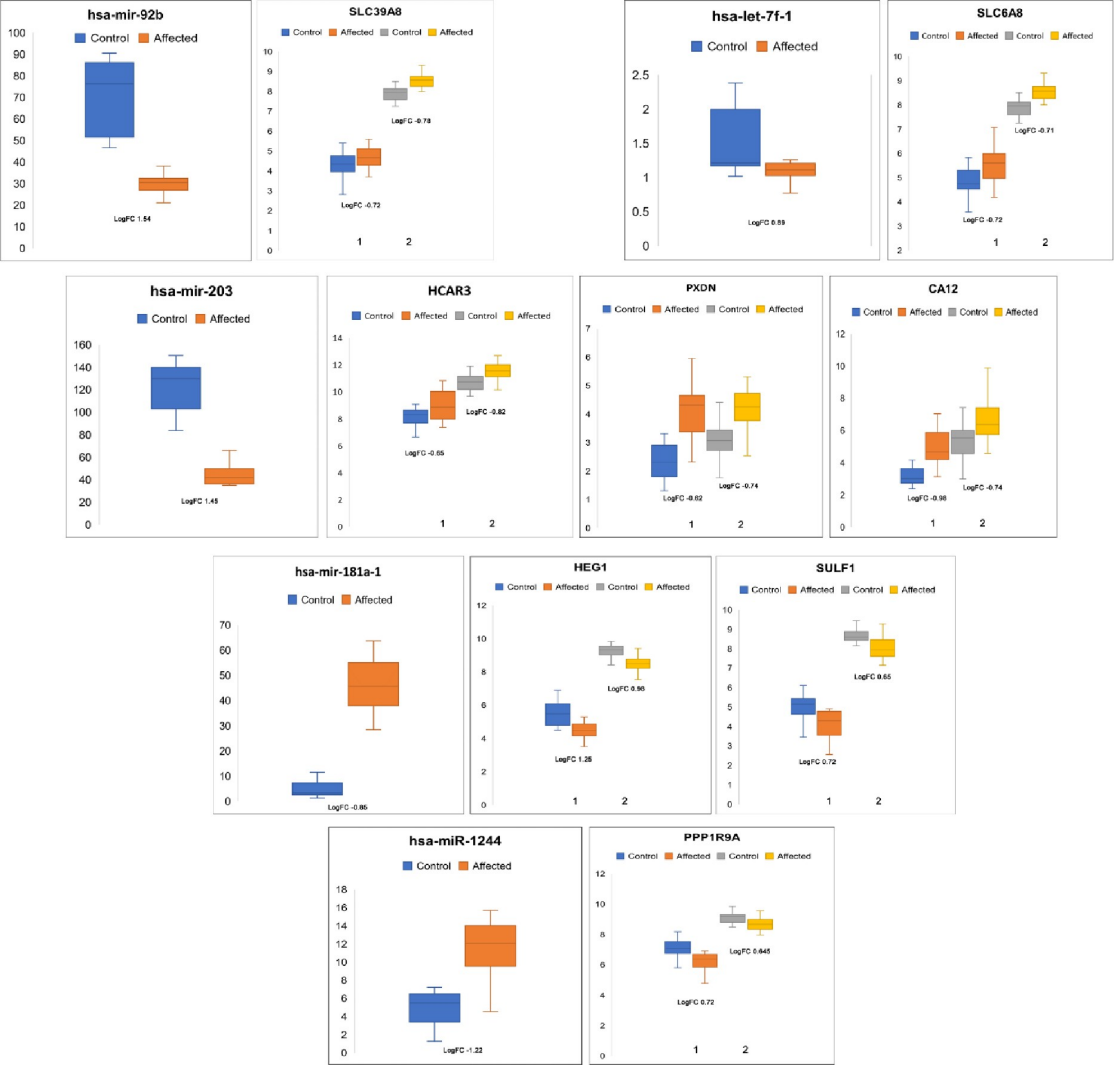
Inverse correlation expression of top four miRNA and its target genes in asthma patients.

We used miRTarBase to obtain the set of validated miRNA-target gene lists that include 380,639 miRNA target gene interactions (MTIs). We combined MTIs of 2,599 miRNAs and 15,064 target genes. Furthermore, we obtained the 41 (out of 71 miRNA) DEMs targeting 7017 genes in the above validated MTIs (**Table 1**). To identify the target-enriched miRNAs and their regulatory roles, we subsequently applied two complementary statistical approaches as described in the methods section. The hypergeometric statistical test revealed that 37 DEMs (15 up- and 12 down-regulated) were inversely correlated (FDR <0.10) with the DEGs, implying miRNA-DEGs functional co-relationship (**Fig 3**).

**Table 1:** The top differentially expressed miRNAs and their target genes based on miRbase target scores.

No.	miRNA Name	Target Gene	Target Rank	Target Score
1	hsa-miR-203a-3p	CAB39	5	99
2	hsa-miR-203a-3p	THSD7A	6	99
3	hsa-miR-335-3p	FUT9	32	99
4	hsa-miR-181c-5p	REPS2	35	99
5	hsa-miR-559	RMND5A	16	99
6	hsa-miR-30d-3p	ROR1	18	98
7	hsa-miR-181c-5p	IL2	54	98
8	hsa-miR-888-5p	CTR9	4	98
9	hsa-miR-203a-3p	CLOCK	28	97
10	hsa-miR-203a-3p	AQP4	29	97
11	hsa-miR-877-3p	KIF5B	11	97
12	hsa-miR-335-3p	ANTXR2	74	97

### miRNA-target gene interaction network

**Fig 4:**
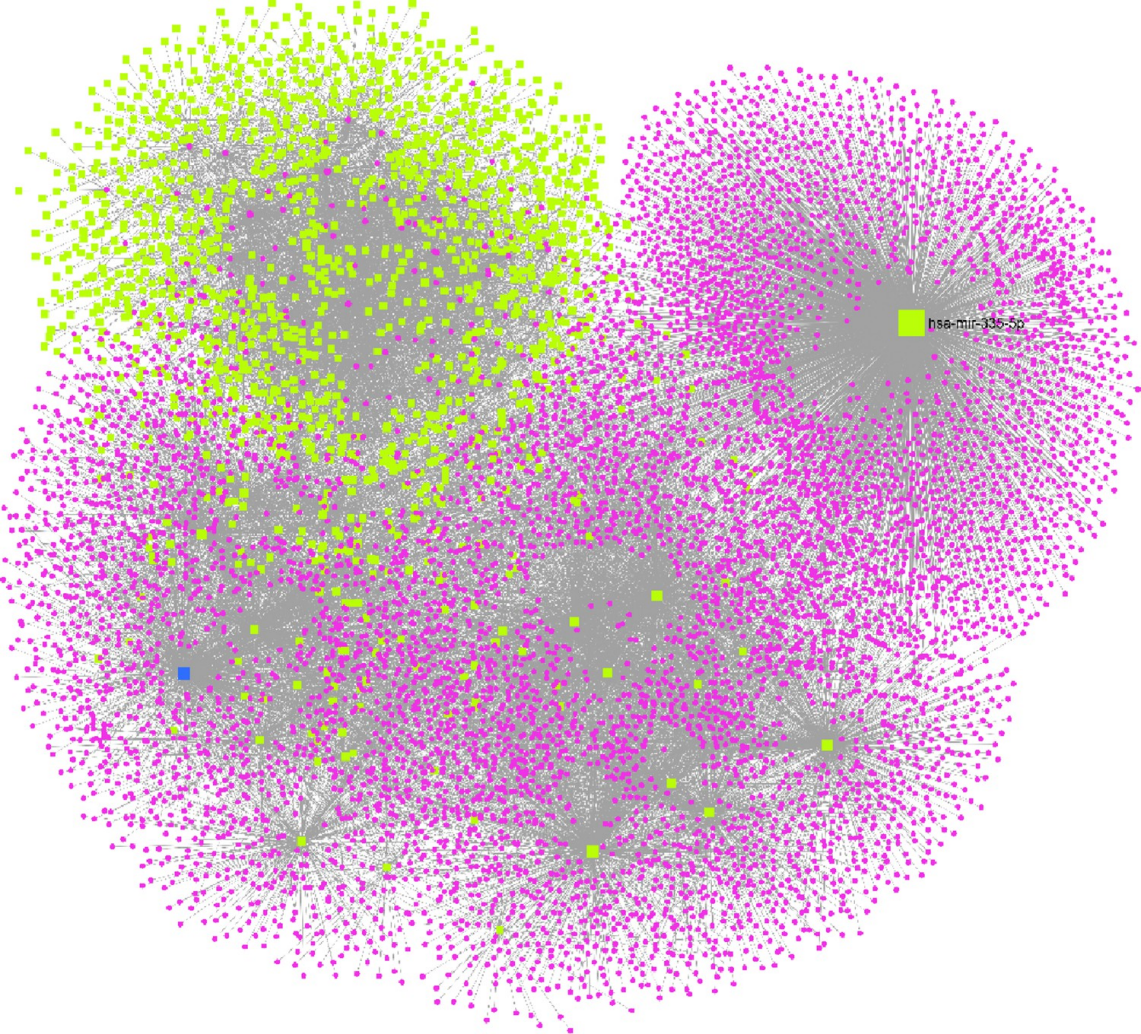
Interaction network between top differentially expressed miRNAs (yellow square) and its target genes (pink circles).

To better understand the role and functions of miRNAs and their target genes, we investigated the miRNA-DEGs protein subnetworks associated with the 37 miRNAs and their inversely correlated target DEGs. A total of 82 nodes and 322 interaction pairs in the PPI-miRNA network were identified (**Fig 4**). In each module, hub miRNAs (module and miRNA p-value > 0.85) were used as input nodes to measure the node localization degree. The miRNA-PI network analysis resulted in four miRNAs (hsa-mir-335-5p, hsa-mir-193b-3p, hsa-mir-181a-5p, and hsa-mir-203a-3p) with a centrality of >315. The hsa-miR-335-5p showed the highest degree of centrality score >2627 with target genes: *ATE1*, *KLF9*, *CA12*, *FHL1*, *NTRK2*, *SCD*, *ST8SIA4*, *CDON*, *PPP1R9A*, *LRRC8A* and *PPP1R3B*. In contrast, hsa-mir-193b-3p, hsa-mir-181a-5p, hsa-mir-203a-3p showed approximate centrality scores of 853, 562, and 315, respectively, and they were found to interact with *LRRC8A*, *CA12*, *ST8SIA4*, *DOCK10*, *NTRK2*, *ABL1*, *ACVR2B*, *AKT2*, and *DLX5* genes **(Table 2)**.

**Table 2:** The miRNA network centrality scores and its target gene pairs.

Label	Degree	Betweenness	Target	Accession	miRNA Seq (5`-3`)	UTR length
hsa-mir-335-5p	2627	7179475.778	ATE1	MIMAT0000765	UCAAGAGCAAUAACGAAAAAUGU	3259
hsa-mir-193b-3p	853	2801907.098	CCND1	MIMAT0002819	AACUGGCCCUCAAAGUCCCGCU	3207
hsa-mir-181a-5p	562	1748817.585	MTF2	MIMAT0000256	AACAUUCAACGCUGUCGGUGAGU	2070
hsa-mir-203a-3p	315	947555.247	DLX5	MIMAT0000264	GUGAAAUGUUUAGGACCACUAG	346
hsa-mir-181a-5p	29	73578.09654	SCD	MIMAT0000256	AACAUUCAACGCUGUCGGUGAGU	3903
hsa-mir-155-5p	18	6132.725	PCDH7	MIMAT0000646	UUAAUGCUAAUCGUGAUAGGGGUU	3952

### Binding affinity between miRNA-mRNA duplex

RNA hybrid webserver was utilized to display significant hybridization between potential viral precursor miRNAs and complementary templates of the potential human miRNAs. Their corresponding minimal free energy of hybridization is given in **Table 3**. The minimal free energy of hybridization was ranged between -15.9 kcal/mol to −33.7 kcal/mol. Based on the sequence similarity, hybridization, and calculated minimum free energy (MFE), five potential miRNAs (hsa-miR-193b-3p, hsa-miR-203a-3p, hsa-miR-335-5p, hsa-mir-155-5p, and hsa-miR-181a-2-3p) were predicted as biomarkers for further analysis of transcriptional factors.

**Table 3:** The miRNA and target gene binding locations and their binding scores.

miRNA	Target Gene	Target Seq	miRNA Seq	MFEs*
hsa-miR-335-5p	ATE1	5′U**GCAU**GXX**U**G**GU**AUUUU**UUGUCUUG**U3′	3′X**UGUA**AAA**A**G**CA**AUXXX**AACGAGAACU5′**	-20.5 kcal/mol
hsa-mir-193b-3p	CCND1	5′A**AGC**A**GGACUUUGA**GGCAAGUGU**GGGCA**CXX3’	3′X**UGC**C**CCUGAAACU**XXXXXXXXX**CCCGGU**CAA5’	-33.7 kcal/mol
hsa-miR-181a-2-3p	SCD	5′XG**U**G**GCUG**UG**GGUGU**GGGUGG**GAGUGU**G3′	3′UG**A**G**UGGC**UG**UCGCA**AXXXXXX**CUUACA**A5′	-26.6 kcal/mol
hsa-miR-181a-2-3p	MTF2	5′XXXA**AU**G**GGCAGC**U**UUGGA**X**GU**A3′	3′UGAG**UG**G**CUGUCG**C**AACUU**A**CA5′**	-21.2 kcal/mol
hsa-mir-203a-3p	DLX5	5′A**UGGUG**CCUUGAAAUCUAU**G**A**CCU**C**AAC**U**UUUCA**A3′	3′G**AUCAC**XXXXXXXXXXXXXX**C**A**GGA**U**UUG**U**AAAGU5′**	-15.9 kcal/mol
hsa-mir-155-5p	PCDH7	5′A**AAUUCC**X**AUU**CUUAAAGAG**GCGGUUAGCA**CXXX3′	3′X**UUGGGG**AUAGXXXXXXXXX**UGCUAAUCGU**AAUU5′	-22.2 kcal/mol

* minimum free energy

### miRNAs-TFs-target regulatory network analyses

**Fig 5:**
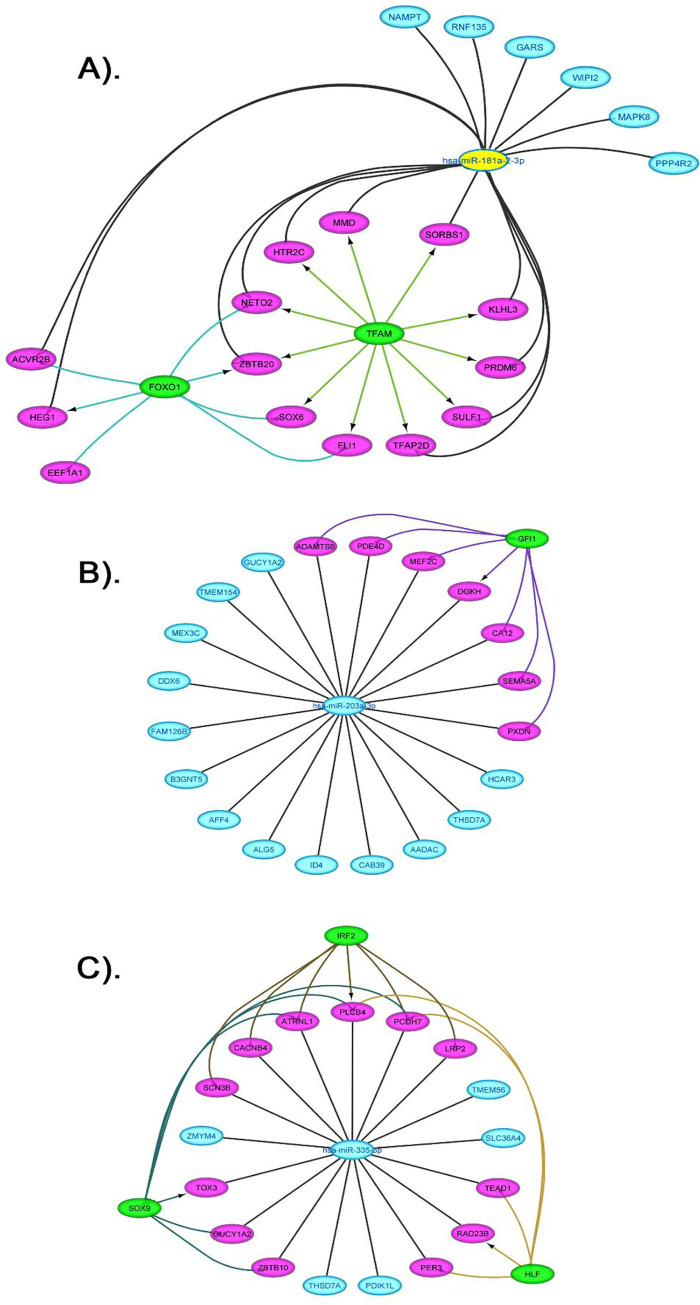
Regulatory networks of the TFs, miRNAs, and target genes. (A) Network of 2 transcription factors and hsa-181a; (B) network of 1 transcription factor and hsa-miR-203a; (C) network of 2 transcription factors and hsa-miR-335. Green color oval represent TFs. Pink ovals represents target genes regulated by miRNAs and TFs, target genes regulated by the miRNAs are represented by blue oval.

To further identify transcriptional factors and regulatory elements of miRNA, we constructed the miRNAs-TFs-target regulatory network using the Cytoscape. The network was constructed using four miRNA (hsa-miR-193b-3p, has-miR-203a-3p, hsa-miR-335-5p, hsa-mir-155-5p and hsa-miR-181a-2-3p) and their target DEGs. Our results showed that hsa-miR-181a-2-3p; hsa-miR-203a-3p; hsa-miR-335-5p have indirect interactions with 6 TFs (*TFAM*, *FOXO1*, *GFI1*, *IRF2*, *SOX9* and *HLF*) and direct interactions with 32 co-regulators (DEGs) (**Fig 5; Table 4**).

**Table 4:** The miRNAs, transcription factors and target gene network enrichment score.

miRNA	# Rank	Motif id	AUC	NES	Cluster Code	Transcription factor	Target genes
hsa-miR-193b-3p	6	transfac_pro-M01099	0.207306	4.4455	M3	RORC, RORB, RORA, GATA6, GATA2, GATA4, GATA3, GATA1, GATA5, YY1	*PLAG1*, *ERBB4*, *FLI1*, *MAPK10*, *KCNJ2*, *FHDC1*
	7	homer-M00057	0.202074	4.31071	M4	FLI1, ETV2, ETV1, ELK4, ELK1, GABPA, ETS1, ETV4, ERF, ETV3, ETV5, ELF4, ETV6, ERG, ETV7, FEV, ELK3, ETS2, EHF, ELF2, ELF3, ELF1, ELF5, GABPB1, SPIB	*FLI1*, *PLAG1*, *LRRC8A*, *RAPGEF6*
	9	hdpi-IRF1	0.199178	4.2361	M6	IRF1	*PLAG1*, *KCNJ2*, *ERBB4*, *MAPK10*, *FLI1*, *LYRM2*
	10	tfdimers-MD00575	0.19404	4.10372	M7	HMGA2, HMGA1, SOX17, ETV7, GABPB1, ETV6, ELF2, ELF4, SPIB, SOX4, ELK4, ELK1, FLI1	*PLAG1*, *DCAF7*, *FLI1*, *ERBB4*, *FHDC1*, *MAPK10*
has-miR-203a-3p	4	hdpi-ZRSR2	0.228635	4.51536	M3	ZRSR2	*-*
	6	tfdimers-MD00426	0.223045	4.37454	M5	GFI1	*-*
	7	transfac_pro-M02271	0.208748	4.01429	M3	HOXA5	*-*
hsa-miR-335-3p	1	hdpi-TSNAX	0.233799	5.66978	M1	TSNAX	*ATRNL1*, *PCDH7*, *PLCB4*, *LRP2*, *TEAD1*
	2	tfdimers-MD00494	0.228436	5.5196	M2	TBP, SOX9	*PCDH7*, *TOX3*, *GUCY1A2*, *PLCB4*, *ATRNL1*, *ZBTB10*
	3	hdpi-HIST2H2AB	0.222896	5.3645	M3	HIST2H2AB	*ATRNL1*, *THSD7A*, *PLCB4*, *ZMYM4*, *SCN3B*, *TEAD1*, *GUCY1A2*, *PCDH7*, *CACNB4*
	8	jaspar-MA0043.1	0.186143	4.33543	M6	HLF, DBP, NFIL3, TEF	*PER3*, *TEAD1*, *PLCB4*, *PCDH7*, *RAD23B*
	10	tfdimers-MD00527	0.184824	4.2985	M7	IRF7, IRF6, ZEB1, IRF4, IRF2, IRF5, IRF8, IRF3, IRF1, POU5F1, IRF9, MYB, E2F1, STAT2, STAT1, SPI1	*PCDH7*, *LRP2*, *CACNB4*, *SCN3B*, *ATRNL1*, *PLCB4*
hsa-miR-181a-2-3p	1	yetfasco-1622	0.157069	4.76021	M1	FOXO1, FOXO3, FOXC2, FOXA2, FOXO4	*ZBTB20*, *FLI1*, *ACVR2B*, *HEG1*, *EEF1A1*, *SOX6*, *NETO2*
	3	hdpi-TFAM	0.145071	4.28686	M3	TFAM, LAS1L	*TFAP2D*, *NETO2*, *ZBTB20*, *MMD*, *FLI1*, *KLHL3*, *SOX6*, *PRDM6*, *SORBS1*, *HTR2C*, *SULF1*
	5	tfdimers-MD00013	0.142554	4.18753	M4	MYB, YY1, TCF4, TAL1, TCF3, TBX5, NR3C1, ZFP42, PGR, MAFA, NR2F2, NR2F1	*TFAP2D*, *PELI2*, *SOX6*, *DGKG*, *ACVR2B*, *SULF1*, *SORBS1*
	6	tfdimers-MD00396	0.137283	3.97957	M5	STAT6, CEBPB, DBP, STAT1, TCF3, ELF2, TCF4, ELK1, ELK4, ETV7, GABPB1, FLI1, ETV6, ELF4, SPIB	*ZBTB20*, *HEG1*, *FLI1*, *SOX6*, *SULF1*, *MMD*, *SORBS1*, *KLHL3*, *PELI2*
	9	hdpi-THAP5	0.133624	3.83523	M8	THAP5	*MMD*, *PRDM6*, *ZBTB20*, *TFAP2D*, *SLC9A5*, *HEG1*
	10	hdpi-LAS1L	0.131028	3.7328	M3	LAS1L, TFAM	*TFAP2D*, *FLI1*, *NETO2*, *ZBTB20*, *MMD*, *KLHL3*, *SOX6*, *PRDM6*, *SULF1*, *SORBS1*, *RAB9B*, *HTR2C*

## Discussion

In the current work, we employed numerous bioinformatic tools to systemically analyze the gene expression data and to identify the regulatory and co-expression networks between the miRNAs and their target gene pairs in asthma. Our functional enrichment analysis showed that most of the DEGs were significantly enriched in ‘response to mineralocorticoid’ under GO- biological processes category [37–41]. It is supported by the fact that, cortisol resistance in asthma conditions has been proposed and the involvement of the 11beta-HSD-2 enzyme has been suggested. Another GO term enriched is integrin, is supported by several studies [42]. A recent report has shown that integrinα7 protein is significantly increased in severe asthma [42]. Similarly, various integrins are shown to have a role in asthma pathophysiology [43]. Interestingly, targeting the integrin α7β1 signaling pathway has been proposed recently as an anti-asthma therapy [42]. In the molecular function category, the term ‘phosphatidylinositol 3-kinase (PI3K)’ was highly enriched which is supported by various studies. Since, PI3K has a central role in in inflammation and hyperresponsiveness of asthma pathophysiology, and hence, it is an attractive molecular target for asthma [44].

The molecular function term, ‘protein phosphatase 1 (PP1)’ was significantly enriched. The PP1 muscle-specific glycogen-targeting subunit (PPP1R3A) is thought to play a role in muscle glycogen regulation and is implicated in asthmatic airway obstruction and hyperresponsiveness. PP1 is a regulatory protein in bronchial smooth muscle that regulates airway hyperresponsiveness (AHR), and it is regulated by protein CPI-17 [45]. Furthermore, fluctuations in CPI-17 signals have been reported to occur in asthma [46]. Interestingly, the role of miR-133a in bronchial smooth muscles (BSM) in the context of PP1 and protein CPI-17 has been reported in asthma pathogenesis [47]. Chemokine activity is another enriched GO term in our analysis and its role is supported by several reports [48]. Various chemokines have been implicated in asthmatic responses. In particular, targeting chemokines and their receptors has been proposed as a new drug target against the asthma [48].

The KEGG pathway annotations of DEGs have revealed the importance of the IL-17 signaling, T helper 1 (Th1) and T helper 2 (Th2) cell differentiation, and Th17 cell differentiation in asthma. The deregulation of important signaling pathways is known to play an important role in a variety of inflammatory diseases. Various studies in human and murine models have suggested the role of IL-17 in airway hyperresponsiveness, while in humans, an increase in IL-17 levels has been observed in asthma [49]. Moreover, it should be noted that the IL17 mediated signaling pathway also regulates mRNA stability. Based on this, we propose future study to understand its role in miRNA-mRNA functional network stability during asthma progression [50].

Another identified pathway is involved in the Th1 and Th2 cell differentiation, whose imbalance causes dysregulation of cytokine profiles [51]. Interestingly, drugs like mangiferin can exert an anti-asthmatic effect by modulating Th1/Th2 cytokine imbalance. Moreover, several let-7 family miRNAs namely, miR-1, mir-19, miR-126, miR-155, and miR-221, that regulate Th2 inflammatory responses by downregulating IL-13 and VEGF, are known for their association with asthma pathogenesis [52]. Our analysis is consistent with the recent reports that Th17 cells play an important role in asthma pathogenesis [53]. A recent study has revealed that hsa-miR-223-3p is a neutrophil-derived microRNA with a prominent regulatory effect on Th17 signaling and endoplasmic reticulum (ER) stress in severe asthma [52]. All these observations conclude that asthma is a chronic airway inflammatory disease characterized by T-helper cell immune responses and other immunological inflammatory responses [52].

The discovery of miRNAs has had a profound effect on the understanding of gene expression and is now considered to be part of the epigenetic machinery. It has led to the addition of a new level of gene regulation, adding a layer of complexity to the central dogma. Due to their gene regulatory functions, miRNAs affect various cellular functions, including cell growth, metabolism, cell death, and animal development. As per miRBase [54], human genome has around 1917 hairpin precursors and 2654 mature miRNAs, several of which have already been implicated in human disease [55]. The miRNAs regulate various signaling pathways in humans and, thus, deregulation of miRNAs can lead to various diseases, including cardiovascular disease, cancers, rheumatoid arthritis and asthma. The multi-target action of miRNAs enables them to regulate the entire signaling network consisting of various signaling molecules, genes, and TFs, thus regulating disease pathology. Mechanistically, miRNA mainly binds to the 3’-untranslated region (3’-UTR) of the target mRNA through imperfect base pairing, which downregulates gene expression or inhibits translation [56]. Similarly, the binding of miRNAs to the 5′ UTR and coding regions of mRNA has silencing effects on gene expression. Interestingly, some studies have also shown miRNAs up-regulate gene expression [57]. Moreover, it is widely known that a single miRNA can target several genes and single gene may be the target of multiple miRNAs, which shows a complex relationship between miRNAs and gene expression [58].

Numerous miRNAs are found to be involved in asthma, which includes downregulated let-7 family, miR-375, miR-193b, as well as upregulated miR-21, miR-223, miR-146a, miR-142-5p, miR-142-3p, miR-146b, and miR-155. Interestingly, most of these miRNAs affect Th2 and Th1 cytokine secretion in the bronchial smooth muscle cells, affecting other inflammatory responses [59]. In severe asthma, miR-221 regulates smooth muscle proliferation and miR-28-5p, and miR-146a/b which activate circulating CD8+ T cells [60]. MicroRNA expression has also been shown to be influenced by inhaled steroids [61]. Taken together, these studies indicate that miRNAs are important regulators of asthma pathogenesis.

Identification of miRNA target genes (mRNA) is a daunting task and has largely been overcome using advanced computational programs [62]. *In silico* prediction is a powerful tool to further validate the results in the absence of appropriate functional data. Indeed, our RNAhybrid webserver-based prediction suggested a stable complex between the miRNA and the corresponding target mRNA (Table 3). The minimal free energy of hybridization ranged from -15.9 kcal/mol to −33.7 kcal/mol which suggests that a stable RNA duplex complex formation is necessary for the miRNA function. The formation of miRNA-mRNA duplexes has a big impact on gene expression and diseases progression. Binding of miRNA may inhibit mRNA translation, leading to gene downregulation. Competition among different miRNA for the target mRNA binding site can also have functional consequences. Similarly, improper RNA folding is also known to alter potential miRNA binding sites, thus affecting normal cellular function that could lead to disease [63, 64]. Furthermore, we also believe that the SNPs affecting the miRNA seed pairing region between the miRNA and the target gene should also be studied as any change in these seed regions can affect the miRNA biogenesis and function as these regions are important for the RNA secondary structure and stability of miRNA-target mRNA pairing. Interestingly, in one study, a SNP in the seed region of miR-499a-3p which was important for the miRNA-mediated silencing mechanism plays contributes to the susceptibility of asthma in children and adolescents with bronchial asthma [65].

Our miRNAs-TFs-target regulatory network analysis has detected the potential miRNAs namely, hsa-miR-193b-3p, hsa-miR-203a-3p, hsa-miR-335-3p, hsa-miR-181a-2-3p and hsa-mir-155-5p (Table 4; Fig 5). Their functional significance in the context of the proposed targets is described. To illustrate, hsa-miR-193b-3p has been reported to be involved in several diseases like leukemia, Amyotrophic Lateral Sclerosis (ALS), and chronic diseases like localized cutaneous leishmaniasis (LCL). It is also differentially expressed in cigarette smokers. However, based on our analysis, its target gene, cyclin D1 (CCND1) is upregulated in asthma serum-sensitized human airway smooth muscle. Moreover, an association of the CCND1 genotype with the asthma susceptibility has been observed [66]. These results suggest that hsa-miR-193b-3p may regulate asthma by regulating the cell cycle [67] (Table 4).

The miR-203a-3p has been reported to be involved in several cancers, which include colorectal cancer [68], hepatocellular carcinoma [69] and bladder cancer [70]. In asthma, miR-203a-3p has been shown to regulate TGF-β1-induced epithelial–mesenchymal transition (EMT) by regulating the SMAD3 signaling pathway [71]. Moreover, downregulation of miR-203a-3p is reported in bronchial epithelial cells and has been suggested to be a potential target for the treatment of asthma [72]. Based on our analysis (Table 4), miR-203a-3p interacts with an IL-4 induced transcription factor (TF) and the DLX5 gene [73]. Notably, IL-4 is a T helper 2 (Th2)-derived interleukin and that Th2 linked inflammatory response is linked to asthma pathogenesis [74], which is consistent with our KEGG pathway findings (Fig 2).

Another miRNA, hsa-miR-335-5p, has been implicated in several cancers, including colorectal cancer [75], gastric cancer [76], and uterine sarcoma [77]. The hsa-miR-335-5p is reported as a biomarker for inflammation related to knee osteoarthritis [78]. ATE1 is a target of hsa-miR-335-5p (Table 4), which is an arginyl-transferase and has been linked to higher metabolic rate and fat [79], while in asthma, obesity and the high body mass index (BMI) are considered as risk factors [80]. These data suggest that hsa-miR-335-5p may regulate ATE1 in obese asthmatic people. Interestingly, obese asthmatics have severe symptoms and poorer response to many asthma medications [81].

The hsa-miR-181a-2-3p has been proposed as a biomarker for sepsis and its associated lung injury [82]. It is involved in several inflammatory responses linked to bronchial and lung tissue [83] and is also increased in the mouse model of asthma [84]. MTF2, a target of hsa-miR-181a-2-3p, has been linked to the PI3K pathway in asthma [85] and is consistent with our GO analysis where the term ‘PI3K’ was highly enriched (Fig 2). Importantly, PI3K role in asthma is well documented [44]. Another target protein, stearoyl-CoA desaturase (SCD) is an important regulator of fat metabolism and is implicated in obesity [86]. Interestingly, reduced levels of SCD and altered fatty acid metabolism have been reported in asthma [87] (Table 4).

The hsa-mir-155-5p is a multifunctional, proinflammatory, oncogenic miRNA that regulates the immune response, chronic inflammation, and autoimmunity [88]. It regulates Th2 cells and hence has a role in asthma; it is altered in severe asthma [89, 90]. The hsa-mir-155-5p targets a well-known asthma gene, PCDH1, which encodes protocadherin-1, which is mainly expressed in the bronchial epithelium and lungs. PCDH1 is essential in the pathogenesis of asthma [91]. Interestingly, two miRNAs identified in our study, hsa-miR-335-5p and hsa-miR-155-5p (Table 4), were associated with long-term lung function change on inhaled corticosteroid (ICS), which is critical in asthma treatment and has prognostic value [89]. Based on our extensive analysis, our data suggests a causal link between the miRNAs’ mediated regulation of target genes in asthma pathogenesis. We sincerely acknowledge the limitations associated with the pooling of analyzed transcripts, low probe specificity, and sample hybridization efficiency factors as we used secondary data from the publicly available databases.

In conclusion, differentially expressed miRNAs and mRNAs that play a potential role in asthma development are identified. The current study presents the functional landscape of human microRNA–mRNA-TF interactions in asthmatics using comprehensive bioinformatic analysis of publicly available microRNA and mRNA expression data sets. This study identified miRNAs (hsa-miR-193b-3p, hsa-miR-203a-3p, hsa-miR-335-5p, hsa-miR-181a-2-3p, hsa-miR-155-5p), which regulate important genes and TFs in asthma pathogenesis. This study is believed to be among the few studies in asthma that use diverse computational analyses to identify miRNA-mRNA and TFs and the functional enrichment of biological pathways involved in asthma. Our results also indicate that genes working at the upstream of a pathway are functionally more important as a minor change in their expression can have a wider effect downstream in any given disease pathology [92]. Better understanding of regulatory networks between disease-causing genes, miRNAs, and TFs, is important for understanding the molecular pathology of asthma. Our study holds the promise of the possible development of novel asthma biomarkers and therapeutic options.
